# Low-Threshold Nanolaser Based on Hybrid Plasmonic Waveguide Mode Supported by Metallic Grating Waveguide Structure

**DOI:** 10.3390/nano11102555

**Published:** 2021-09-29

**Authors:** Xin Zhang, Meng Yan, Tingyin Ning, Lina Zhao, Shouzhen Jiang, Yanyan Huo

**Affiliations:** 1 Shandong Provincial Key Laboratory of Optics and Photonic Device & Shandong Provincial Engineering and Technical Center of Light Manipulations, School of Physics and Electronics, Shandong Normal University, Jinan 250358, China; 2019020511@stu.sdnu.edu.cn (X.Z.); 2020020554@stu.sdnu.edu.cn (M.Y.); ningtingyin@sdnu.edu.cn (T.N.); lnzhao@sdnu.edu.cn (L.Z.); 107145@sdnu.edu.cn (S.J.); 2 Collaborative Innovation Center of Light Manipulations and Applications, Shandong Normal University, Jinan 250358, China

**Keywords:** nanolaser, hybrid plasmonic waveguide mode, low-threshold, *Q*-factor

## Abstract

A high *Q*-factor of the nanocavity can effectively reduce the threshold of nanolasers. In this paper, a modified nanostructure composed of a silver grating on a low-index dielectric layer (LID) and a high-index dielectric layer (HID) was proposed to realize a nanolaser with a lower lasing threshold. The nanostructure supports a hybrid plasmonic waveguide mode with a very-narrow line-width that can be reduced to about 1.79 nm by adjusting the thickness of the LID/HID layer or the duty ratio of grating, and the *Q*-factor can reach up to about 348. We theoretically demonstrated the lasing behavior of the modified nanostructures using the model of the combination of the classical electrodynamics and the four-level two-electron model of the gain material. The results demonstrated that the nanolaser based on the hybrid plasmonic waveguide mode can really reduce the lasing threshold to 0.042 mJ/cm^2^, which is about three times lower than the nanolaser based on the surface plasmon. The lasing action can be modulated by the thickness of the LID layer, the thickness of the HID layer and the duty cycle of grating. Our findings could provide a useful guideline to design low-threshold and highly-efficient miniaturized lasers.

## 1. Introduction

Nanolaser based on the surface plasmon (SP) can generate a coherent high-intensity field beyond the diffraction limit [1,2], with applications in ultrasensitive sensing, spectroscopy, nonlinear optics, ultrafast on-chip sources, etc. [3,4,5,6,7,8]. However, the high radiative and ohmic loss of surface plasmon results in the higher lasing threshold. Various methods were proposed to reduce the laser threshold, such as designing new nanostructures, using new materials and so on [9,10,11,12,13]. High *Q*-factor indicates that the microcavity has a strong ability to restrain light and produce a higher density of states, which could enhance the interaction between light and matter, thus reducing the threshold of laser interaction [14].

Multi-layer films consisting of metal and dielectric waveguide layers can effectively reduce the loss and improve the *Q* factor [15,16]. In such a system, the coupling between the SP and the waveguide mode (WG) can create a hybrid resonance mode with narrow line-width and high *Q* factor. To obtain a low-loss hybrid mode, various plasmonic structures have been studied. For example, a multilayer dielectric under the metallic arrays can support a narrow-band transmission peak with only a few nanometers [17,18]. Li et al. proposed narrow transmission dips with the line-with of 8 nm in the dielectric grating on the semiconductor–metal–semiconductor stacks [19]. Liu et al. came up with a periodic plasmonic structure, which supports a resonance with line-width only 3 nm [20]. These resonance modes have potential applications in sensing, optical filtering, and optoelectronic devices integration because of their narrow line-width and high *Q* factor [21,22].

The hybrid resonance mode can also be used in the nanolaser, because it can reduce the loss effectively by concentrating the mode inside the dielectric waveguide layer, and effectively reduce the threshold of the nanolaser. In 2009, the Xiang Zhang group reported a plasmonic laser using a hybrid plasmonic waveguide that can localize the transmission mode in the micro-cavity formed by the gap layer, so the loss in the metal can be greatly reduced [23]. Degl’Innocenti et al. demonstrated the realization of the terahertz quantum cascade laser based on a hybrid plasmonic waveguide [24]. In recent years, the lasing threshold was reduced using the hybrid plasmonic waveguide mode of the nanowire waveguide mode [23,24,25,26,27,28], and the threshold of some nanolasers can be reduced to 0.2 mJ/cm^2^ [27].

In this paper, we investigated a hybrid plasmonic system composed of a silver grating and low-index dielectric layer (LID) coated on high-index dielectric layer (HID), and it supports a hybrid plasmonic waveguide mode with very narrow line-width and high *Q* factor. After we added a gain material layer composed of rhodamine 6G (R6G) onto this hybrid system, we discussed the lasing action of the nanolaser based on the hybrid plasmonic system. The results revealed that the lasing threshold of the nanolaser can be reduced to about 0.042 mJ/cm^2^ that is about three times lower than the nanolaser based on the surface plasmon and even lower than the previously double-layer grating dielectric waveguide structure nanolasers [29]. Furthermore, we found that the *Q* factor can be tuned by adjusting the thickness of the LID layer, the thickness of the HID layers and the grating duty ratio. Therefore, the lasing action can also be tuned by changing these structural parameters.

## 2. Materials and Methods

**Figure 1:**
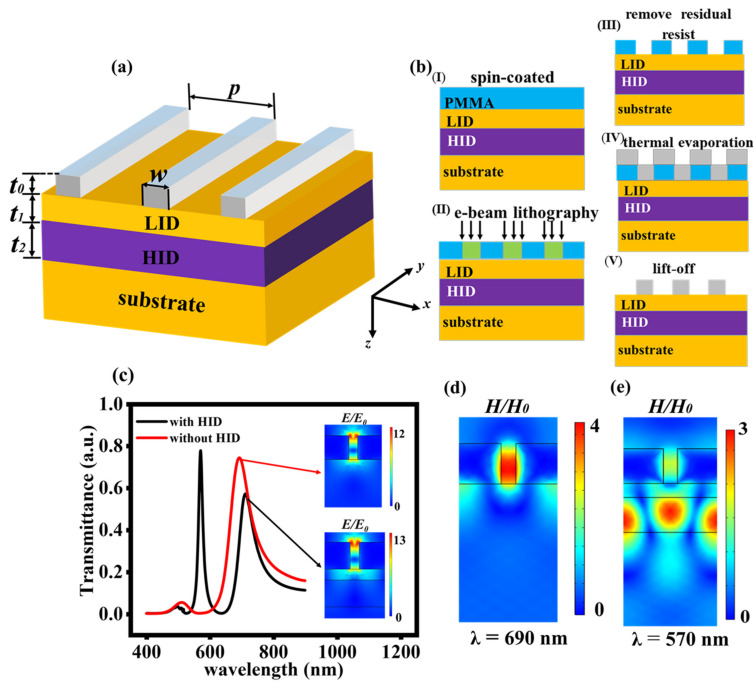
(**a**) Structure diagram of the metallic grating with HID layers on substrate. (**b**) Schematic of the fabrication process using e-beam lithography. (**c**) Transmission spectra of the structures (*t*_0_ = 150 nm, *t*_1_ = 60 nm, *t*_2_ = 150 nm and *D* = 0.855) with and without HID layers. Insets: Electric field distribution of nanostructures at two wide transmission peaks. (**d**,**e**) Magnetic field distribution of nanostructures at wide (*λ* = 690 nm) (**d**) and narrow transmission peak (*λ* = 570 nm) (**e**).

When LID and HID layers were introduced under a metallic nanograting (M–LH nanostructure), a resonance mode with narrower transmission peak can be obtained [30], and it is schematically displayed in Figure 1a. The refractive index of the LID layer and the substrate was set as *n*_1_ = 1.5, such as SiO_2_. The refractive index of HID layer was *n*_2_ = 2.0, such as TiO_2_ [31]. The metal was chosen as silver, and the dielectric permittivity of silver was defined by the Lorentz–Drude model, $\varepsilon_{Ag}=1-\omega^{2}{}_{p}/\left(\omega^{2}+i\gamma \omega \right)$, where $\omega_{p}$ and γ represent the plasma frequency and damping frequency of silver [32]; *t*_0_*, t*_1_ and *t*_2_ are the thickness of grating, LID and HID layer; *p* marks the grating period; *w* means the grating ridge width and the grating duty cycle *D* refers to the ratio of *w* to *p*. The finite-difference time-domain method (FDTD) was adopted to calculate the spectral characteristics and the lasing performance of the nanostructures in the following calculations. We selected one period (*p* = 345 nm) as the simulation domain. Bloch–Floquet periodic boundary conditions were imposed to *x* direction of the unit cell. In the *z* direction, perfectly matched layer (PML) boundary conditions were applied on the boundary of the simulation domain. A plane-wave light of transverse magnetic (TM) polarization incident normally onto the M–LH nanostructures.

Figure 1b illustrates schematically the fabrication process of the M–LH nanostructures. Firstly, we deposited an HID layer on SiO_2_ substrates by using magnetron sputtering equipment. Then the LID layer was sputtered onto the HID/SiO_2_ substrates. To fabricate the grating structure by e-beam lithography, a PMMA layer (positive resist) was spin-coated on the LID/HID/SiO_2_ substrate to form a resist layer. Secondly, the e-beam lithography was carried out to fabricate a grating structure using a grating pattern. Thirdly, we removed any residual resist on the substrate. Fourthly, the Ag film was deposited by thermal evaporator. Finally, Ag grating structures were obtained by a lift-off process.

Figure 1c shows the transmission spectrum of the metal grating with HID layer (black solid line) and without HID layer (red solid line). When there is no HID layer underneath the grating, only a wide peak appears at *λ* = 690 nm. It is a SP mode that possess high loss. Its magnetic field mainly distributes in grating layer as shown in Figure 1d. However, when the HID layer is added underneath the grating, a narrow transmission peak appears at *λ* = 570 nm compared with the wide nearly-null transmission of the metal nanoarrays without the HID layer. We investigated the magnetic field distribution of the resonance mode at narrow transmission peak in Figure 1e. It is a hybrid SP waveguide mode [30], and its magnetic field mainly distributes in HID layer, which leads to less loss. We used this hybrid mode to realize nanolaser with a lower threshold. For the wide peak at *λ* = 702.6 nm, it is still a SP mode. The resonant wavelength redshifts to 702.6 nm because of the addition of the HID layer. The intensity of the wide transmission peak decreases to 0.6 because the transmitted electric field is affected by the HID layer, as shown in the insets of the Figure 1c. 

## 3. Results

### 3.1. Effects of Structural Parameters on Transmission Spectrum

**Figure 2:**
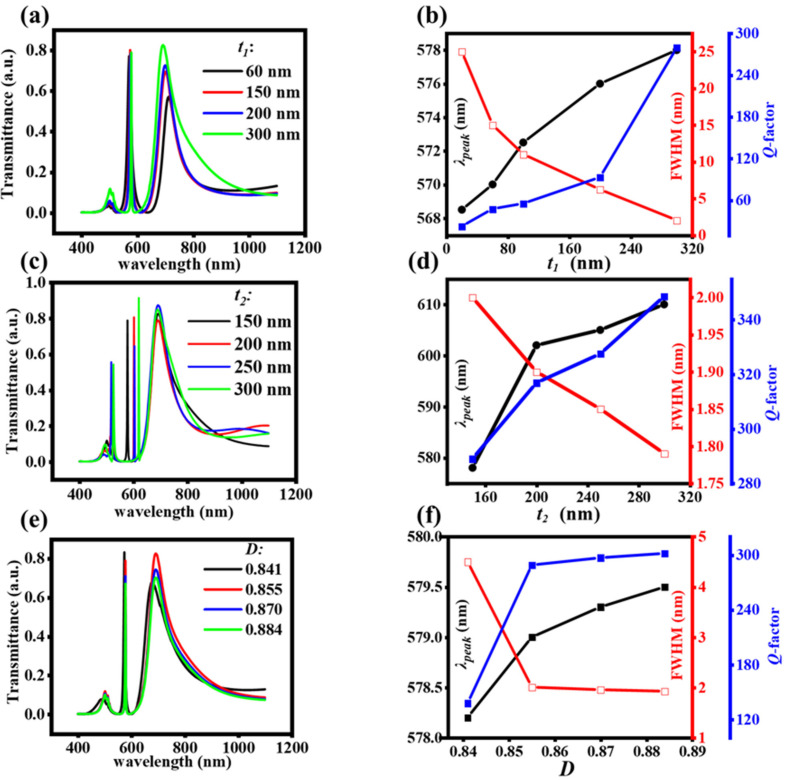
Effect of *t*_1_, *t*_2_ and *D* on transmission spectra. (**a**,**c**,**e**) Transmission spectra of the structure with different *t*_1_, *t*_2_ and *D*. (**b**,**d**,**f**) The *λ*_peak_, FWHM and *Q*-factor as the function of *t*_1_ (**b**), *t*_2_ (**d**) and *D* (**f**), respectively.

The full width at half maximum (FWHM) of the narrow transmission peak is affected by the thickness of the LID and HID layers [17,30,31,33,34,35], and it also can be affected by the grating duty cycle. In this part, we investigated the roles and effects of *t*_1_, *t*_2_ and *D* on the narrow transmission peak in Figure 2. Figure 2a shows the transmission spectra of the M–LH nanostructure with different *t*_1_*,* the thickness of HID layer *t*_2_ remains at 150 nm, and the grating duty cycle *D* remains at 0.855. As *t*_1_ increases, the position of the narrow transmission peak red shifts slightly and the FWHM becomes smaller. Figure 2b displays the dependence of the wavelength (*λ*_peak_), the FWHM and the *Q* factor of the narrow transmission peak on different *t*_1_. The *Q* factor is calculated by *Q* = *λ*/Δ*λ*, *λ* is the wavelength of the resonance mode, Δ*λ* is the FWHM of the transmission peak. The wavelength red shifts only from 568.2 to 578 nm, the line-width decreases from 25 to 2.0 nm, and the *Q*-factor increases from 22.7 to 289 as *t*_1_ increases from 20 to 300 nm. Figure 2c,d illustrate the influence of the thickness of the HID layer *t*_2_ on the narrow transmission peak. We chose the optimal *t*_1_ of 300 nm and *D* keeps 0.855. The wavelength of the narrow transmission peak red shifts a lot and the FWHM also becomes smaller when *t*_2_ increases. When *t*_2_ increases from 150 to 300 nm, the wavelength red shifts from 570 to 610 nm, the line-width decreases only from 2 to 1.79 nm, and the *Q*-factor increases from 289 to 348. If we choose *t*_1_ = 60 nm, the line-width of the narrow peak can decrease from 15 to 3.3 nm, and the *Q*-factor increases from 38 to 190 as *t*_2_ increases from 150 to 300 nm. Besides, we found that other peaks appear at smaller wavelengths, such as the peak at *λ* = 538 nm and *λ* = 542 nm for *t*_2_ = 250 and 300 nm in Figure 2c, which are the hybrid SP higher-order waveguide modes. The effects of the grating duty cycle *D* on the narrow peak are exhibited in Figure 2e,f, the thickness of the LID keeps 300 nm and HID layer all keeps 150 nm. The wavelength of the narrow transmission peak remains almost the same, while the FWHM becomes smaller with the increase in *D*. When *D* increases from 0.841 to 0.884, the line-width decreases from 4.2 to 1.8 nm, and the *Q* factor increases from 137.6 to 321.1. 

### 3.2. The Reasons of the Change of the Narrow Transmission Peak

**Figure 3:**
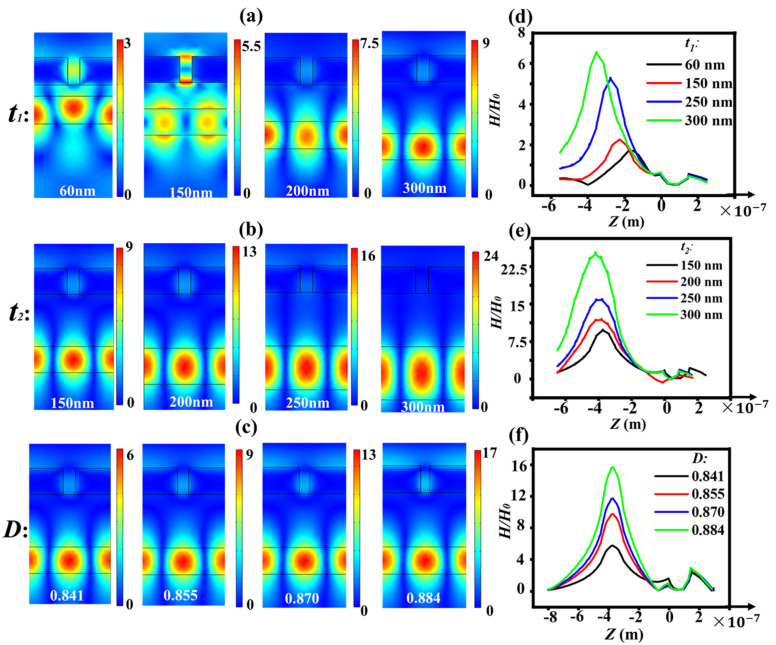
(**a**–**c**) The magnetic field distribution of the nanostructure with different *t*_1_ (**a**), *t*_2_ (**b**) and *D* (**c**). (**d**–**f**) Displays the magnetic field variation curve of the nanostructure at the boundary (*x* = 172.5 nm) with the variation of *t*_1_ (**d**), *t*_2_ (**e**) and *D* (**f**). The bottom of the metal grating locates at *z* = 0.

In order to investigate the reasons of the change of the narrow transmission peak with different *t*_1_, *t*_2_ and *D*, we plotted the field distributions (*H/H*_0_) of the M–LH nanostructures with different *t*_1_, *t*_2_ and *D* in Figure 3. Figure 3a exhibits the magnetic field distributions of the structure with different *t*_1_. With the increase in the thickness of LID *t*_1_, the field is more localized into the high refractive index layer. Figure 3d shows the comparison of the fields along *x* = 172.5 nm in Figure 3a. The intensity of the field localized in the HID layer increases gradually from 3 to 8.8. The thickness of the LID layer affects the strength of the coupling between the SP mode and waveguide mode. The larger the *t*_1_ is, the weaker the coupling is. The more field localizes into the HID layer, the weaker field at the interface of metal-LID layer is (as shown in Figure 3d), which means that the metal loss will be weakened. Therefore, the line-width of the narrow transmission peak becomes narrower, and the *Q* factor increases. As the position of the narrow peak mainly depends on the HID layer, the redshifts slightly are caused by the weaker coupling. 

**Figure 4:**
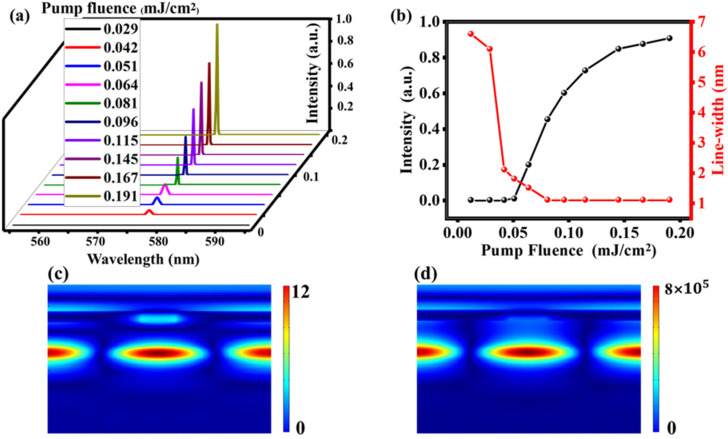
Lasing actions of the hybrid M–LH system with *t*_1_ = 300 nm, *t*_2_ = 140 nm and *D* = 0.884. (**a**) Normalized emission spectra of the hybrid M–LH nanostructures as a function of pumping fluence. (**b**) Emission spectra line-width and the maximum intensity as a function of pump fluence. (**c**,**d**) Field distributions when pump fluence is 0.029 mJ/cm^2^ and 0.191 mJ/cm^2^.

Figure 3b,e show the field distributions of the structure with different *t*_2_. With the increase in the thickness *t*_2_, the field localized into the HID layer increases from 8.6 to 25, and the field at the interface metal-air decrease, thus weakening the metal loss. However, the field localized in the metal slit increases with the increase in *t*_2_, and thus will increase the metal loss. So, the changes of line-width and *Q* factor with *t*_2_ are not as obvious as that with *t*_1_. The red shift of the narrow peak is caused by the increase in the effective volume of the HID cavity with the increase in *t*_2_, because the resonance wavelength of nanocavity is proportional to the effective volume of the cavity [36]. Figure 4c,f illustrates the field distributions of the structure with different *D*. With the increase in the duty cycle *D*, the field localized into the HID layer increases from 5.5 to 16.1, and also weakens the metal loss. Thus, with an effective adjustment of metal loss, an optimized lasing action with low threshold can be realized.

### 3.3. Lasing Action

We choose rhodamine 6G (R6G) as the gain medium, and it is described using a four-level energy two-electron model. The dynamics of the population densities of every level can be derived from the rate equation model, as shown below [37]:

(1) $$\frac{dN_{3}}{dt}=-\frac{N_{3}}{\tau_{32}}-\frac{N_{3}}{\tau_{30}}+\frac{1}{\hbar \omega_{30}}\cdot \vec{E}\cdot \frac{d\vec{P_{30}}}{dt}\;\frac{dN_{2}}{dt}=\frac{N_{3}}{\tau_{32}}-\frac{N_{2}}{\tau_{21}}+\frac{1}{\hbar \omega_{21}}\cdot \vec{E}\cdot \frac{d\vec{P_{21}}}{dt}\;\frac{dN_{1}}{dt}=\frac{N_{2}}{\tau_{21}}-\frac{N_{1}}{\tau_{10}}-\frac{1}{\hbar \omega_{21}}\cdot \vec{E}\cdot \frac{d\vec{P_{21}}}{dt}\;\frac{dN_{0}}{dt}=\frac{N_{3}}{\tau_{30}}+\frac{N_{1}}{\tau_{10}}-\frac{1}{\hbar \omega_{30}}\cdot \vec{E}\cdot \frac{d\vec{P_{30}}}{dt}$$

The four levels involved in laser generation include ground state level *E*_0_, excited state high energy level *E*_3_, laser upper level *E*_2_ (metastable energy level) and laser lower level *E*_1_. *N*_0_, *N*_3_, *N*_2_ and *N*_1_ are electron population density in each of the four energy levels. *τ_ij_* is the decay time between levels *i* and *j*. $\vec{E}$ represents the total electric field, which can be calculated by solving the Maxwell equations. $\vec{P_{21}}$ and $\vec{P_{30}}$ correspond to the net macroscopic polarization associated with the emission and absorption transitions from level 2 to level 1 and level 3 to level 0, and are coupled with the electromagnetic fields. $\vec{P_{21}}$ and $\vec{P_{30}}$ satisfy the following equation [38]:

(2) $$\frac{d^{2}\vec{P_{21}}}{dt^{2}}+{\;\gamma }_{21}\frac{d\vec{P_{21}}}{dt}\;+{\;\omega }_{21}^{2}\vec{P_{21}}=\;\xi_{21}\left(N_{2}-N_{1}\right)\vec{E}\;\frac{d^{2}\vec{P_{30}}}{dt^{2}}+\;\gamma_{30}\frac{d\vec{P_{30}}}{dt}+\omega_{30}^{2}\vec{P_{30}}={\;\xi }_{30}\left(N_{3}-N_{0}\right)\vec{E}$$

where ω_21_ is the transition frequencies between level 2 and level 1, ω_30_ means the transition frequencies between level 3 to level 0. $\;\gamma_{21}$ and $\gamma_{30}$ stand for the full-widths at half-maximum of these transitions from level 2 to level 1 and level 3 to level 0. $\xi_{21}=6\pi \varepsilon_{0}c^{3}/\omega_{21}^{2}\tau_{21}$, $\xi_{30}=6\pi \varepsilon_{0}c^{3}/\omega_{30}^{2}\tau_{30}$. By coupling Equations (1) and (2), the amplification process of the nanolaser can be solved by the FDTD method. In this work, the energy level parameters of R6G are obtained from the reference [11], $\tau_{30}$ = 1 ns, $\tau_{21}$ = 3 ns,$\;\tau_{10}$ = $\tau_{32}$ = 50 fs, $\gamma_{30}\;$ = 3.26 × 10^14^ rad/s, $\gamma_{21}=$ 1.54 × 10^14^ rad/s, $\omega_{30\;}$ = 3.68 × 10^15^ rad/s and $\omega_{21\;}$ = 3.30 × 10^15^ rad/s. Furthermore, the total molecular density is of *N*_0_ = 3.8 × 10^24^, and is roughly calculated by the concentration of the solution prepared in experiment.

When a gain material layer composed of R6G and PVA is covered on the hybrid M–LH nanostructure, the lasing action is investigated in Figure 4. We use Lumerical’s FDTD Solutions software to study the lasing action. In the beginning of the simulation, all of the dye molecules are in the ground state, *N*_0_ = 1. We then use a 4 ps pump pulse, centered at 512 nm, to excite the molecules. The pump is linearly polarized along the *y*-axis. In order to ensure lasing action, the simulation time was set to 30 ps, the mesh size was set 2 nm. Note that, the resonance wavelength of the narrow peak red shifts to 590 nm when PVA layer is covered on the M–LH system. In order to make the resonance wavelength of the narrow peak match the emission peak of R6G, we adjusted the structure parameters of the system to: *D* = 0.884, *t*_1_ = 300 nm and *t*_2_ = 140 nm. The normalized emission spectrum of the hybrid M–LH nanostructure as a function of the incident pump fluence are shown in Figure 4a. Figure 4a illustrates the normalized emission spectra of the hybrid M–LH nanostructures as a function of the pumping fluence. At first, the intensity of the narrow peak increases slowly as the pump fluence increases, and it is still a broad spectrum. However, when the pump fluence increases to 0.042 mJ/cm^2^, the intensity of the narrow peak suddenly increases and the spectrum suddenly becomes sharper (the line-width of the emission spectra reduced from 6.7 nm to only 1.2 nm), which are the typical characteristics of stimulated emission behavior of a laser system. This pump fluence is the threshold of the nanolaser. The normalized maximum emitted intensity and the line-width of the narrow peak as a function of the input pump fluence are plotted in Figure 4b, and it also clearly displays the threshold of the nanolaser. The threshold of the nanolaser based on this modified structure is not as low as the Perovskite nanoribbon based nanolaser on plasmonic grating [38,39], but lower than the nanolaser double waveguide dielectric grating [29]. When the pump fluence is below and above the threshold, we plotted the magnetic field distribution of the hybrid plasmonic system in Figure 4c,d. When the pump energy is greater than the threshold, the intensity of *H/H*_0_ of the narrow peak can be amplified about 6.6 × 10^4^ times compared to the intensity of *H/H*_0_ of the nanostructure with no gain.

**Figure 5:**
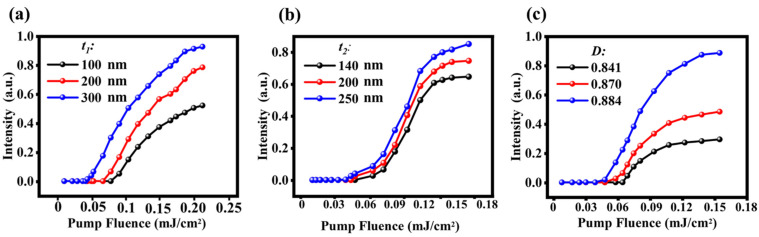
Maximum emission intensity as function of the pump fluence with different *t*_1_, *t*_2_ = 140 nm and *D* = 0.855 (**a**), with different *t*_2_, *t*_1_ = 300 nm and *D* = 0.855 (**b**), and with different *D, t*_1_ = 300 nm, *t*_2_ = 140 nm (**c**).

The *Q* factor and the line-width of the narrow peak in hybrid plasmonic system can be adjusted by *t*_1_, *t*_2_ and *D,* as shown in Figure 2, which will affect the lasing action of the nanolaser. We investigated the lasing action of the nanolaser based on our system with different *t*_1_, *t*_2_ and *D*. The maximum emission intensity as a function of pump influence is plotted in Figure 5. It can be seen that every curve has the same characteristics of stimulated emission behavior. Nevertheless, the lasing thresholds and the emission intensity are different. It is obvious that the lasing threshold value decreases from 0.072 to 0.045 mJ/cm^2^ and the maximum emission intensity increases as *t*_1_ increases from 100 to 300 nm, as exhibited in Figure 5a. According to Figure 5b, the lasing threshold value decreases only from 0.048 to 0.043 mJ/cm^2^ (because the *Q* factor changes very little with *t*_2_ when *t*_1_ = 300 nm) and the maximum emission intensity also increases as *t*_2_ increases from 200 to 300 nm. The lasing threshold and the maximum emission intensity also can be adjusted by the duty cycle *D.* When *D* increases from 0.841 to 0.884, the lasing threshold decreases from 0.068 to 0.042 mJ/cm^2^ and the maximum emission intensity also increases. The *Q* factor increases, and the metal loss decreases with the increase of *t*_1_, *t*_2_ and *D*, which are caused by the more field localized into the HID layer and the weaker field at the interface of metal-LID layer. Note that, the resonant wavelength of the spectrum will change along with the variation of *t*_2_. In order to confirm the influence of the change of structural parameters on the laser behavior, we set $\omega_{21}$ at the resonant frequencies of each structure in Figure 5b, and other parameters of gain material are the same as R6G.

**Figure 6:**
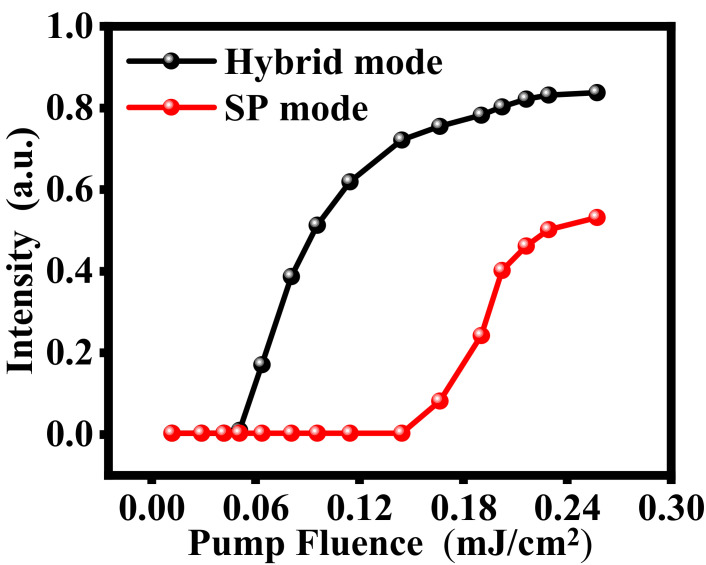
Comparison of thresholds of nanolasers based on the hybrid plasmonic SP waveguide mode (black line) and the SP mode (red line) of the M–LH structure (*t*_1_ = 300 nm, *t*_2_ = 140 nm and *D* = 0.881).

In order to further clarify that the threshold of the nanolaser based on the hybrid SP waveguide mode of the metallic grating on LID/HID layers can indeed be reduced, we compared the threshold of nanolasers based on the hybrid SP waveguide mode and the SP mode. when *t*_1_ = 300 nm, *t*_2_ = 140 nm and *D* = 881, there still have a broad transmission peak at 723.4 nm like Figure 1b that is a SP mode. To compare the effects of different modes on the laser action, we set $\omega_{21}$ at the respective resonant frequencies of the two modes, and other parameters of gain material are the same as R6G. Figure 6 shows the comparison of the lasing action of the nanolaser based on the hybrid SP waveguide mode and the SP mode. We discovered that the threshold of the nanolaser based on the SP mode has a higher threshold about 0.145 mJ/cm^2^. The maximum emission intensity is also lower than the nanolaser based on the hybrid plasmonic waveguide mode. The SP mode has very low *Q* factor only about 10.0 that is caused by the high metal loss. 

It should be noted that although there are two peaks when the HID layer is added, multiple modes will not occur when we study the lasing behavior of the hybrid SP waveguide mode. Firstly, the lasing mechanism of the nanolaser [10] operates as follows: the pump light pumps electrons to the high energy level *E*_3_, then relaxes to the laser upper level *E*_2_ (metastable energy level). When the electrons transfer down from *E*_2_ to *E*_1_, the energy is coupled to the hybrid SP waveguide mode excitation by near-field radiationless transitions, then the mode is amplified to form the lasing. So, the wavelength of the hybrid SP waveguide mode must match the energy level difference between *E*_2_ and *E*_1_, i.e., the resonance wavelength of the narrow peak must match the emission peak of R6G. Secondly, the threshold of the nanolaser based on the SP mode is larger than the nanolaser based on the hybrid SP waveguide mode. Therefore, we can control the energy of the pump light so that only one mode appears during lasing.

## 4. Conclusions

In summary, we explored the lasing behavior of a nanolaser based on a nanostructure by introducing LID/HID layers underneath the metallic grating. The M–LH nanostructure supports a narrow transmission peak with very narrow line-width (in a scale of tens of nanometers down to only 1.79 nm) and very high *Q* factor (up to 348). According to analysis, it is a hybrid SP waveguide mode formed by coupling with the SP mode and traditional waveguide mode. When a gain medium composed R6G is covered on the M–LH nanostructure, the lasing actions of nanolaser based on our modified structure using the four-level two-electron system were investigated. The results proved that the lasing threshold of nanolaser can be reduced to 0.042 mJ/cm^2^, and the lasing action can be adjusted by changing the structural parameters of the modified structure.

## Data Availability

Data is contained within the article.
